# Wireless Sensor-Based Smart-Clothing Platform for ECG Monitoring

**DOI:** 10.1155/2015/295704

**Published:** 2015-11-12

**Authors:** Jie Wang, Chung-Chih Lin, Yan-Shuo Yu, Tsang-Chu Yu

**Affiliations:** ^1^School of Software Technology, Dalian University of Technology, Dalian 116620, China; ^2^Department of Computer Science and Information Engineering, Healthy Aging Research Center, Chang Gung University, Taoyuan 33302, Taiwan

## Abstract

The goal of this study is to use wireless sensor technologies to develop a smart clothes service platform for health monitoring. Our platform consists of smart clothes, a sensor node, a gateway server, and a health cloud. The smart clothes have fabric electrodes to detect electrocardiography (ECG) signals. The sensor node improves the accuracy of QRS complexes detection by morphology analysis and reduces power consumption by the power-saving transmission functionality. The gateway server provides a reconfigurable finite state machine (RFSM) software architecture for abnormal ECG detection to support online updating. Most normal ECG can be filtered out, and the abnormal ECG is further analyzed in the health cloud. Three experiments are conducted to evaluate the platform's performance. The results demonstrate that the signal-to-noise ratio (SNR) of the smart clothes exceeds 37 dB, which is within the “very good signal” interval. The average of the QRS sensitivity and positive prediction is above 99.5%. Power-saving transmission is reduced by nearly 1980 times the power consumption in the best-case analysis.

## 1. Introduction

As the population is becoming progressively older, higher quality of health and medical care is now expected in countries with significant aging problems. This care is particularly important for countries with 75% of the elders suffering from chronic diseases [1]. According to a recent statistical analysis in 2012, two million deaths are caused by heart and cerebrovascular diseases every year in China [2]. Continuous recording of biomedical signals by smart devices is critical for the advancement of diagnosis as well as the treatment of cardiovascular diseases. For the elders, wearable devices, such as smart clothes, enable early detection through long-term trend analysis to limit the occurrence of acute events and chronic cardiovascular diseases [3].

Wireless and sensor technologies facilitating noninvasive sensors integrated into clothing can enhance disease prevention [4–6]. Axisa et al. designed and developed smart and flexible sensors for healthcare and illness prevention [7]. Jovanov et al. [8] proposed a wireless body area network using different types of sensing units, such as electrocardiography (ECG), body tilt, pulse oximetry (SpO_2_), and knee activity. The sensor data are sent via Bluetooth to mobile devices and are routed to nursing homes or workstations via the Internet. MIThril [9] is a next-generation, wearable research platform developed by researchers at the MIT Media Lab. The MIThril hardware platform combines body-worn computation, sensing, and networking in a clothing-integrated design. The MIThril software platform is a combination of user interface elements and machine learning tools built on the Linux operating system. Nguyen et al. [10] designed a body sensing module, integrating the optical linear encoder (OLE) and an accelerometer. A sensor network of three sensing modules was established via a controller area network bus to capture human arm motion. For heart condition monitoring, the LifeShirt system is accurate in detecting both heart rate and heart rate variability [11]. Recent studies have found more evidence that heart rate variability is associated with mild and severe depression [12, 13], stable chronic obstructive pulmonary disease [14, 15], and attention deficit hyperactivity disorder (ADHD) in children [16].

Recent technologies enable smart clothing to be miniaturized, power-saving, integrated, and comfortable [17, 18]. By combining cloud technology with smart clothing [19], the measured personal physiological data can establish a health database to promote telemedicine and medical services. However, smart clothes still face some bottleneck problems. (1) Continuity: most smart clothes cannot be made as comfortable as ordinary clothes. There are also issues, such as washability, frequent charging, and online updating. (2) Reliability: individual differences and artificial interference can reduce the accuracy of ECG signal extraction, which is crucial for health analysis and disease diagnosis. (3) Power consumption: smart clothes sensors without an intelligent transmission algorithm can result in a huge amount of garbage data being sent to the cloud, consuming an enormous quantity of power.

Taking the above issues into consideration, we present a smart-clothing platform for ECG acquisition, analysis, and transmission for the detection of abnormal ECG. (1) We design washable and comfortable smart clothes using fabric electrodes that connect to the sensor node directly. Thus, the sensor node employs a wireless network to enable noninvasive monitoring of cardiovascular condition. (2) The ECG detection is comprised of a filtering phase and a pattern matching phase. Most normal ECG can be filtered out in the filtering phase, and the remaining abnormal ECG is further analyzed. Morphology analysis is proposed to improve the detection accuracy of QRS complexes. In addition, a reconfigurable finite state machine (RFSM) is defined to support online updating. (3) The power-saving transmission strategy can smartly decide whether to transmit statistical results or raw data depending on the short-term analysis of heart rate variability (HRV) [20–23]. Therefore, power consumption and garbage data can be largely reduced, which is of great significance for telemedicine and medical service.

## 2. Methodology

**Figure 1:**
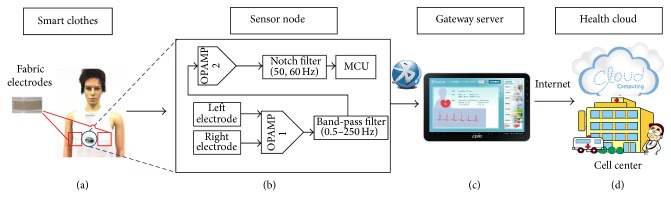
System architecture: (a) smart clothes, (b) sensor node, (c) gateway server, and (d) health cloud.

The overall system architecture is shown in Figure 1. The smart-clothing platform consists of (a) smart clothes with fabric electrodes to collect the ECG signal, (b) a sensor node integrating a signal processing circuit and microprocessor module that can convert biomedical signals to high quality ECG data, (c) an android tablet acting as a gateway server [24] to display the analysis results, and (d) a health cloud service that can transfer personal data to the hospital for health consultation. The smart clothes are mainly composed of fabric electrodes and a sensor node. The smart clothes are washable and have physical properties similar to ordinary clothing. Fabric electrodes are used to obtain lead II ECG signals on the chest. Signals are transmitted to the sensor node via conductive fiber, which is particularly well-adapted for the monitoring of chronic cardiovascular disease.

### 2.1. Sensor Node Design and ECG Analysis Algorithm for Ambulatory QRS Detection

The circuit of the sensor node is shown in Figure 1(b). It contains amplifiers, filters, analog-to-digital converters, MCU, and a Bluetooth module. Because the raw signals are too weak and distorted, an amplifier (100 gain) is required to amplify the differential signal and constrain the in-phase signal. A band-pass filter (0.5 Hz to 250 Hz) is used for reduction of noise beyond the ECG signal band. Equations (1)–(3) illustrate how to determine the resonant frequency (*F*_*r*_) for the corresponding circuit. *F*_*h*_ and *F*_*l*_ are the maximum cutoff frequency (250 Hz) and minimum cutoff frequency (0.5), respectively. *Q* is a quality factor to characterize a resonator's bandwidth relative to *F*_*r*_. The higher the *Q*, the narrower and sharper the peak. A secondary amplification (68.5 gain) is applied and a notch filter (50 Hz, 60 Hz) is needed for the rejection of a DC component to enhance the AC component. The notch filter is used to reject the 50 Hz and 60 Hz signals, and the capacitance is 0.1 *μ*F. Equation (4) helps to select a proper resistor (*R*) and capacitance (*C*). *F*_notch_ is 60 Hz and *C* is 0.1 *μ*F. The resistor is approximately 26.5 kΩ. The filtered signals are gathered into the microcontroller through an ADC at 1000 Hz. The microcontroller in the sensor node is mainly responsible for analyzing the ECG raw data and controlling the Bluetooth module to transmit the results to the gateway server. Consider

(1) bandwidth=Fh−Fl,

(2) Fr=FhFl,

(3) Q=Frbandwidth,

(4) Fnotch=12πRC.

### 2.2. Ambulatory QRS Detection

**Figure 2:**
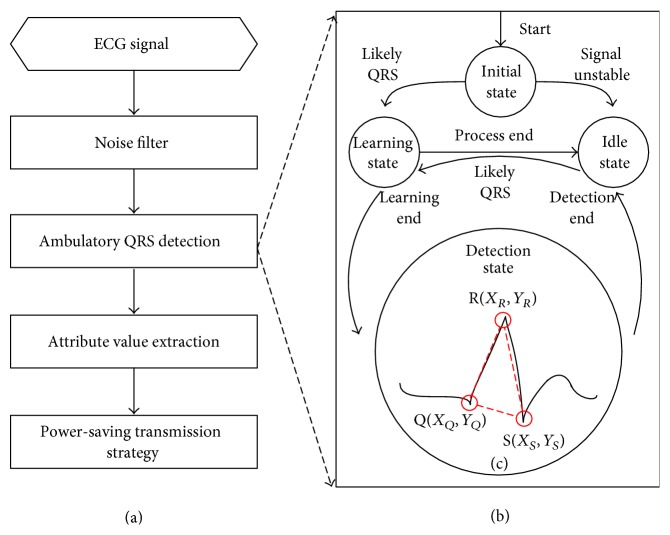
Ambulatory ECG analyzing framework on the sensor node: (a) ECG processing strategy, (b) ambulatory QRS detection procedures, and (c) QRS morphology analysis algorithm.

**Figure 3:**
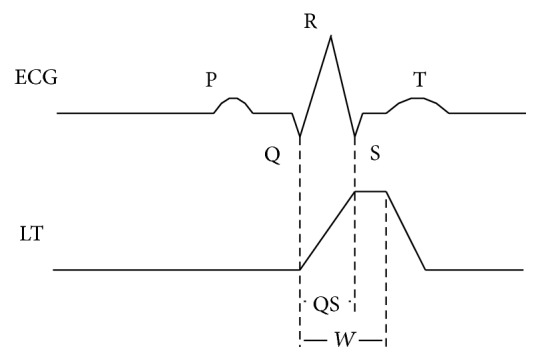
Relationship between the QRS complex and the LT signal [22].

As shown in Figure 2(a), the processing strategy is introduced to analyze raw ECG data. A digital filter and QRS detection algorithm (including ambulatory QRS detection algorithm (Figure 2(b)) and a QRS morphology analysis algorithm (Figure 2(c))) are applied to obtain ECG segmentations in the sensor node. Next, more attribute values of the HRV parameters are calculated according to user requirements. The attribute values are compressed for transmission to the gateway server by our power-saving transmission strategy. The ambulatory QRS detection procedures (illustrated in Figure 2(b)) are described as follows:

(1) Initial State: a low-pass filter [25] and nonlinearly scaled curve length transformation [26] are employed to enhance the QRS complexes and to suppress other parts of the ECG and noise. Equation (1) illustrates the curve length transformation LT (*w*, *i*). Δ*y* is the length differentiable over the time window *w*, and Δ*t* is the sampling period. The time window is chosen to be approximately equal to the QRS duration (*w* = 0.13 second) to calculate the ECG curve length corresponding to the QRS complexes. *i* is the start index. We calculate the curve length transformation from *i* − *w* to *i*. After the length transformation, the Initial State is set to the Idle State if no valid ECG signal is detected. Otherwise, the Initial State goes to the Learning State if a likely QRS complex is detected**:**
  (5) $$\begin{array}{c} \text{LT}\left(\;w,i\;\right)=\overset{i}{\underset{k=i-w}{\sum }}\sqrt{\Delta t^{2}+\Delta y_{k}^{2}}. \end{array}$$
 After the curve length transformation calculation, the location of the QRS complex is indicated by the local maximal curve length.  Figure 3 shows the relationship between the QRS complex and the LT signal.
(2) Idle State: the process in the Idle State is to the low power model until a valid ECG signal is detected. If a likely QRS complex is detected, the state jumps to the Learning State.
(3) Learning State: if the sensor node finds more than 5 likely QRS complex events in 10 seconds, we calculate the signal baseline and switch to the Detection State. If not, the process is interrupted and is returned to the Idle State.
(4) Detection State: a novel QRS morphology analysis algorithm called MWqrs is proposed to differentiate between QRS complexes and artifacts. Three feature points are calculated after the algorithm detects a possible QRS complex. These three points include the QRS onset (named Q point), QRS peak (named R point), and QRS end (named S point) [25]. The coordinates of the three points are Q(*X*_*Q*_, *Y*_*Q*_),  R(*X*_*R*_, *Y*_*R*_), and S(*X*_*S*_, *Y*_*S*_), which form a triangle, as illustrated by the red dotted lines in Figure 2(c). The morphology of this triangle is then analyzed based on biomedical standards.  Table 1 illustrates all the morphology parameters and their ranges based on the biomedical standards. For instance, we define the “R peak” to be the *X*_*R*_ in the range of (*X*_*Q*_, *X*_*S*_). The QRS complex is recognized as a true QRS complex if every morphology parameter is within the min and max range. Otherwise, the complex is annotated as an artifact. If continuous QRS complex errors are detected by our morphology analysis method, the process jumps to the Idle State and notifies the user to adjust the smart clothes to reduce the movement artifact.

### 2.3. Power-Saving Transmission Strategy

**Figure 4:**
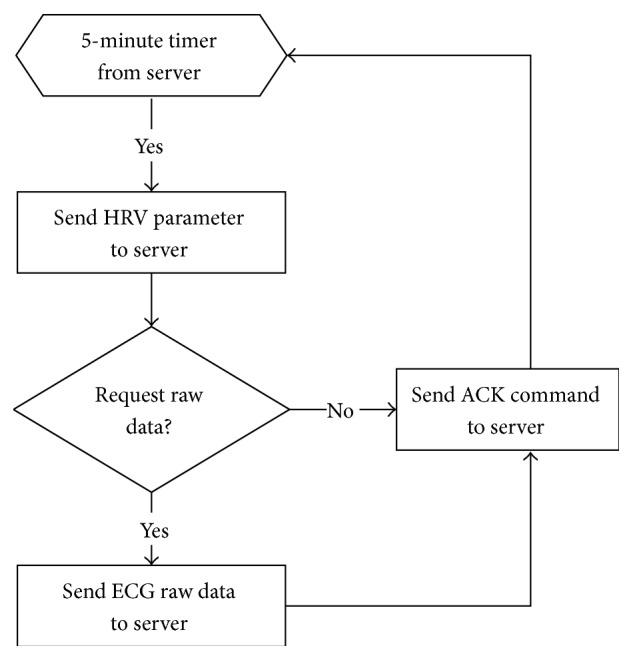
Power-saving transmission data transmission flow.

**Figure 5:**
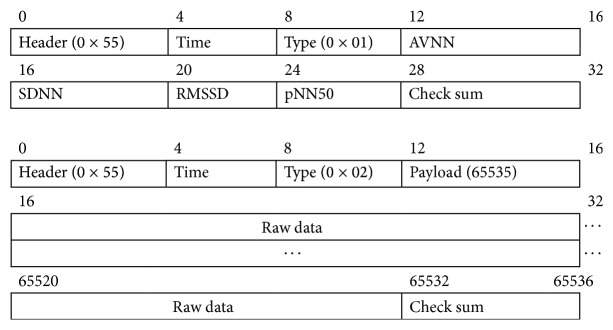
Date package format.

Power-saving transmission is an intelligent transmission strategy designed to compress the ECG data (shown in Figure 4). The gateway server sends a message to the sensor node every 5 minutes to require a statistical value of the past ECG data. The sensor node replies to the gateway server with the acknowledged package and HRV statistical parameters. According to the HRV attribute values, the sensor node sends the raw data if a ECG QRS complex waveform is detected. The package formats of the HRV attribute values and ECG raw data are defined in Figure 5. The first two rows contain the HRV statistical parameters package consisting of 32 bytes, and the subsequent rows contain the ECG raw data package made up of 65536 bytes.

### 2.4. Multipattern Abnormal Disease Matching in Gateway Server Design

Clinicians usually calculate HRV parameters, such as SDNN (standard deviation of all normal RR intervals), LF (low frequency, 0.04–0.15 Hz), and ratio of LF/HF (high frequency, 0.15–0.4 Hz) [20] as attribute values when they want to distinguish subjects of different severity of depression [12]. HRV parameters generate a series of attribute values (Attribute_1_, Attribute_2_,…, Attribute_*n*_) according to the range of disease symptoms. To increase the flexibility of abnormal ECG symptom recognition, an RFSM is proposed to support online updating in the gateway server. The RFSM is used to divide the segmented ECG signals into normal patterns and abnormal patterns depending on the range of the attribute values.

**Figure 6:**
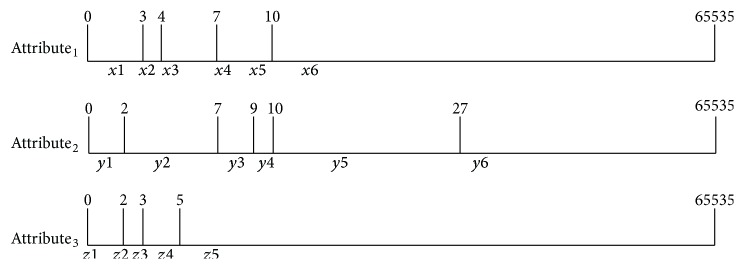
Attribute values indicated by character string.

For ease of explanation, we assume that there are seven patterns, named P1 to P7. These are defined by three attributes (*X*, *Y*, *Z*). The range of each attribute values is from 0 to 65536, and “*∗*” means that the value of this attribute does not relate to this pattern. Each pattern corresponds to an action that indicates a certain type of disease symptom or process (as shown in Table 2). There are several steps to building an RFSM. Firstly, as shown in Figure 6, a series of attribute values (*X*, *Y*, *Z*) are transformed into distributed ranges signed by a character string according to the ranges of patterns (i.e., 0 < *X* < 3 as *X*1, 4 < *X* < 7 as *X*3, *X* = 7 as *X*4, and 7 < *X* < 10 as *X*5). Therefore, all attributes can be illustrated by combined character strings. Secondly, different patterns (P1, P2,…, P7) are converted to regular expressions by the attribute character string. A pattern that matches a certain regular expression is equivalent to a pattern that matches a series of attributes. Seven patterns can be expressed as P1[(*x*1)(*y*2∣*y*3∣*y*4)(*z*5)], P2[(*x*2∣*x*3∣*x*4∣*x*5∣*x*6)(*y*5∣*y*6)(*z*2)], P3[(*x*2∣*x*3∣*x*4∣*x*5∣*x*6)(*y*5∣*y*6)(*z*1∣*z*2∣*z*3)], P4[(*x*3∣*x*4∣*x*5∣*x*6)*z*1], P5[*x*4], P6[*x*6(*y*3∣*y*4∣*y*5∣*y*6)], and P7[(*y*4∣*y*5)*z*1]. Finally, all regular expressions are transmitted to the Deterministic Finite Automaton (DFA) to construct a look-up table [27]. The look-up table is used for the matching of the finite state machine (FSM). Once new patterns are added, the look-up table is regenerated and downloaded to flash memory via the Internet. When the ECG data are ready for analysis, it is transformed into a series of attribute values represented by a character string. When the matching starts, the process jumps to different states in the look-up table according to the character string values, such as (State *i*, String *Xi*) → (State *j*). The matching process continues until it jumps to the final state to match a certain pattern and execute an action.

## 3. Experimental Design and Result

### 3.1. Signal Quality Analysis and Experimental Result

**Figure 7:**
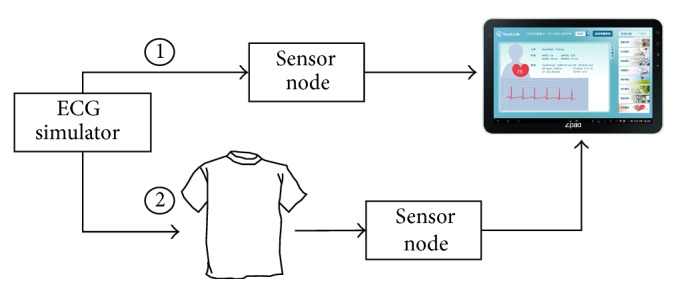
Experimental design flowchart.

Signal quality is a vital factor that guarantees the accuracy of the platform. A criterion named SNR (SNR ≈ 10 log_10_⁡ (Signal Voltage/Noise Voltage)^2^) is introduced to assess the performance of the smart clothes sensor.  Figure 7 illustrates the experimental design of the Signal Voltage and Noise Voltage measurement. (1) Signal Voltage is directly measured from a KL-79106 ECG simulator through the sensor node in different beats per minute (BPM). (2) Noise Voltage is the difference of the raw data with and without the smart clothes sensor. The SNR of different BPM (BPM 60, BPM 80, BPM 100, and BPM 120) is 37.75, 37.52, 37.62, and 37.91 dB, respectively. After calculation of the RR interval by the gateway server, the accuracy of the BPM values with smart clothes is greater than 99.60%. This result shows that the signal quality of smart clothes is very good [28, 29].

### 3.2. QRS Detection Algorithm Experimental Result

The performance of our algorithm is evaluated using the MIT database [30] by comparing the MWqrs algorithm with the Wqrs algorithm [25]. We chose 18 normal subjects from the MIT-BIH Normal Sinus Rhythm Database to verify the algorithm performance. Two indicators, namely,* sensitivity* and* positive prediction*, are introduced to quantify the QRS detection accuracy. The sensitivity parameter indicates how likely it is that the test can detect the presence of a QRS complex, and the positive prediction parameter indicates how likely someone with a QRS complex will show a positive test result. The annotation file (atr) in the MIT database is chosen as the standard comparison. Sensitivity and positive prediction are calculated using the “Bxb” programs from the PhysioToolkit [30]. The experimental results are shown in Table 3. The result shows that the accuracy mostly exceeds 99%, and the average accuracy of the QRS sensitivity is greater than 99.68%. Subject 16272 is the worst case with interference from several artifacts. The positive prediction of the MWqrs algorithm is 95.26%, which is 5.47% higher than the Wqrs algorithm. This shows that our MWqrs algorithm performs better in the presence of ECG artifacts.

To verify the performance of the algorithm for normal and abnormal subjects, we analyzed 18 normal subjects (MIT-BIH Normal Sinus Rhythm Database) and 48 abnormal subjects (MIT-BIH Arrhythmia Database). We calculated the HRV features, including AVNN (the average of all normal RR intervals), SDNN, RMSSD (the root mean square of the differences of successive RR intervals), and pNN50 (the percentage value of consecutive RR intervals that differ by more than 50 ms) [31]. The features we calculated were compared with HRV information calculated from the annotation file in the MIT-BIH database. The average result, best case, and worst case are presented in Table 4. The average accuracy of the HRV features of the abnormal subjects is more than 96%. The accuracy of the best case is higher than 98% and that of the worst case is over 93%. The average accuracy of the normal subjects is better than the abnormal subjects. These experimental results show that the MWqrs algorithm is able to robustly detect ambulatory QRS complexes. In our HRV time domain results, we observe some differences between normal and abnormal subjects; that is, the AVNN, SDNN, and pNN50 values of normal subjects are higher than the abnormal subjects, and the RMSSD values of the normal subjects are lower than the abnormal subjects. These results can be used as rules to decide if raw data are to be transmitted or not.

### 3.3. Power-Saving Transmission Efficacy Analysis

We compared the transmission efficacy every 5 minutes according to different abnormal subject ratios with “full transmission” and “power-saving transmission.” In the power-saving transmission mode, data is transmitted to the gateway server in a 32-byte package at a five-minute interval. In the full transmission mode, data is transmitted continuously at a one-second interval. In total, the size of continuous data transmitted over five minutes is 57,652 bytes (12 bits *∗* 128/sec *∗* 300 s + 20 bytes of package field + 32 bytes of HRV result). A 24-hour data set is emulated using 288 5-minute data sets. The normal ratio is defined as the percentage of normal HRV results analyzed by the multipattern abnormal disease matching in the gateway server. Once the HRV parameters are transmitted to the gateway server, they are analyzed to decide whether to transmit the raw data. For instance, if the normal ratio is 80%, the 24-hour power-saving transmission transmits 288*∗*32  bytes + 57652  bytes*∗*288*∗*(1 − 80%) = 3.176 Mb, and the efficiency is 15.84 Mb/3.176 Mb = 4.99. Suppose that 100% normal patterns can be detected from the subjects; the transmission efficacy is then 1980 times. This is very useful for the analysis of a huge amount of personalized ECG data in the health cloud (Table 5).

## 4. Conclusions

In this paper, we present a smart-clothing platform for ECG acquisition, analysis, and transmission. This platform is dedicated to the detection of abnormal ECG. A washable, low-power consumption, and comfortable smart cloth is designed using fabric electrodes. The SNR values are more than 37 dB, which demonstrates that the smart clothes have very good signal acquisition capability. The reconfigurable firmware architecture enables online updating. In addition, the normal ECG detection process comprises a filtering phase and a pattern matching phase. Both the MWqrs detection algorithm and parameters of the whole platform have more than 99.5% accuracy performance in QRS detection and BPM calculation. The power-saving transmission strategy can smartly compress raw data depending on short-term analysis of HRV, which helps to achieve more than 500 times the transmission efficacy and power reduction.

Our platform can be used for the long-term monitoring of elders' cardiovascular status with high accuracy, simplicity, and future expandability. Our future efforts will focus primarily on abnormal ECG pattern recognition with multichannels. Therefore, the platform can provide clinically meaningful information to doctors to reduce the harm caused by cardiovascular disease.

## Figures and Tables

**Table 1:** QRS morphology parameters.

Indicator	Formula	Min	Max
QRS width	|*X*_*S*_ − *X*_*Q*_|	40 ms	160 ms
QRS height	|*Y*_*R*_ − *Y*_*S*_|	0.05 mV	1.5 mV
*Q* horizontal	|*Y*_*Q*_ − *Y*_*S*_|	0	1.5 mV
RR interval	|*R*_*i*_ − *R*_*i*−1_|^*∗*^	250 ms	1500 ms

^*∗*^ *R*_*i*_ is the real-time R peak position. *R*_*i*−1_ is the previous R peak position of the real-time R peak position.

**Table 2:** Date package format.

Pattern	*X*	*Y*	*Z*	Action
P1	0 < *X* < 3	2 < *Y* < 10	5 < *Z* < 65535	Disease 1
P2	3 < *X* < 65535	11 < *Y* < 65535	*Z* = 2	Disease 2
P3	3 < *X* < 65535	11 < *Y* < 65535	0 < *Z* < 3	Disease 3
P4	4 < *X* < 65535	*∗*	0 < *Z* < 2	Disease 4
P5	*X* = 7	*∗*	*∗*	Artifact
P6	10 < *X* < 65535	7 < *Y* < 65535	*∗*	Alarm
P7	*∗*	9 < *Y* < 27	0 < *Z* < 2	Disease 4

**Table 3:** Normal database test results.

Subjects	QRS sensitivity		Positive prediction	
	Wqrs	MWqrs	Wqs	MWqrs
16256	*100.00%*	99.99%	99.74%	*99.76%*
16272	97.00%	95.76%	89.79%	95.26%
16273	99.99%	99.93%	99.93%	99.98%
16420	99.98%	99.87%	99.79%	99.93%
16483	99.98%	99.93%	99.88%	99.97%
16539	99.97%	99.87%	99.79%	99.94%
16773	99.99%	99.95%	99.96%	99.99%
16786	100.00%	100.00%	99.97%	99.98%
16795	99.99%	99.80%	99.87%	99.95%
17052	99.98%	99.87%	99.52%	99.71%
17453	99.98%	99.80%	99.72%	99.91%
18177	99.98%	99.91%	99.63%	99.74%
18184	99.99%	99.91%	99.55%	99.91%
19088	100.00%	99.95%	98.29%	98.38%
19090	99.99%	99.96%	99.70%	99.80%
19093	100.00%	99.99%	99.87%	99.88%
19140	100.00%	99.99%	99.82%	99.85%
19830	99.93%	99.84%	98.74%	99.07%

Average	99.82%	99.68%	99.09%	99.50%

**Table 4:** HRV time domain analysis results.

Indicator	Normal subjects			Abnormal subjects		
	Average	Best case	Worst case	Average	Best case	Worst case
	(*n* = 18)	# 16256	# 16273	(*n* = 48)	# 230	# 105
AVNN (ms)	787.7	795.5	818.9	747.4	794.9	748.8
Accuracy (%)	99.1%	99.5	98.9	98.4	99.9	96.2

SDNN (ms^2^)	136.5	169.8	135.7	55.3	47.8	62.5
Accuracy (%)	98.9	99.8	98.0	96.7	97.9	93.1

RMSSD (ms^2^)	27.9	39.9	46.2	43.5	61.3	24.2
Accuracy (%)	98.7	99.3	96.9	97.0	98.1	93.7

pNN50 (%)	7.5	17.2	17.9	5.7	9.2	2.8
Accuracy (%)	98.4	99.6	96.3	97.7	98.7	96.7

**Table 5:** Power-saving transmission efficacy results.

Normal ration	0%	20%	40%	60%	80%	100%
Full transmission (Mb/24-hour)	15.84	15.84	15.84	15.84	15.84	15.84

Power-saving transmission (Mb/24-hour)	15.84	12.67	9.54	6.33	3.176	0.008

Efficiency (times)	1	1.25	1.66	2.50	4.99	1980
